# Protective Effect of Dual-Strain Probiotics in Preterm Infants: A Multi-Center Time Series Analysis

**DOI:** 10.1371/journal.pone.0158136

**Published:** 2016-06-22

**Authors:** Luisa A. Denkel, Frank Schwab, Lars Garten, Christine Geffers, Petra Gastmeier, Brar Piening

**Affiliations:** 1 Institute of Hygiene and Environmental Medicine, Charité Universitätsmedizin Berlin, Berlin, Germany; 2 Department of Neonatology, Charité Universitätsmedizin Berlin, Berlin, Germany; University British Columbia, CANADA

## Abstract

**Objective:**

To determine the effect of dual-strain probiotics on the development of necrotizing enterocolitis (NEC), mortality and nosocomial bloodstream infections (BSI) in preterm infants in German neonatal intensive care units (NICUs).

**Design:**

A multi-center interrupted time series analysis.

**Setting:**

44 German NICUs with routine use of dual-strain probiotics on neonatal ward level.

**Patients:**

Preterm infants documented by NEO-KISS, the German surveillance system for nosocomial infections in preterm infants with birth weights below 1,500 g, between 2004 and 2014.

**Intervention:**

Routine use of dual-strain probiotics containing *Lactobacillus acidophilus* and *Bifidobacterium* spp. (Infloran) on the neonatal ward level.

**Main outcome measures:**

Incidences of NEC, overall mortality, mortality following NEC and nosocomial BSI.

**Results:**

Data from 10,890 preterm infants in 44 neonatal wards was included in this study. Incidences of NEC and BSI were 2.5% (n = 274) and 15.0%, (n = 1631), respectively. Mortality rate was 6.1% (n = 665). The use of dual-strain probiotics significantly reduced the risk of NEC (HR = 0.48; 95% CI = 0.38–0.62), overall mortality (HR = 0.60, 95% CI = 0.44–0.83), mortality after NEC (HR = 0.51, 95% CI = 0.26–0.999) and nosocomial BSI (HR = 0.89, 95% CI = 0.81–0.98). These effects were even more pronounced in the subgroup analysis of preterm infants with birth weights below 1,000 g.

**Conclusion:**

In order to reduce NEC and mortality in preterm infants, it is advisable to add routine prophylaxis with dual-strain probiotics to clinical practice in neonatal wards.

## Introduction

Preterm infants weighing less than 1,500 g, very low birth weight (VLBW) infants, represent a very vulnerable group of newborns. Among them, infants with births weight less than 1,000 g constitute the subgroup of extremely low birth weight (ELBW) infants. Both, VLBW and in particular ELBW infants, are at a high risk to develop life-threatening complications such as necrotizing enterocolitis (NEC) and bloodstream infections (BSI) [1].

NEC is the most common complication of the gastrointestinal tract in VLBW infants [2–4]. Data from NEO-KISS, the German national surveillance system for nosocomial infections in VLBW infants, reported 962 (2.9%) cases of NEC among 33,048 VLBW infants between 2007 and 2011 [5]. The frequency of NEC, however, varies by country and neonatal intensive care unit (NICU) [2]. In German NICUs, NEC is associated with a high attributable mortality of 14.7% [6]. Nosocomial BSI is one of the most frequent complications of VLBW infants. 5,735 cases of nosocomial BSI (17.4%) among 33,048 VLBW infants were observed by NEO-KISS between 2007 and 2011 [5]. The attributable mortality of BSI in German NICUs was calculated 1.4% [6]. Thus, due to the high frequency of BSI and high attributable mortality of NEC in preterm infants, prevention of these complications should be of high priority.

Probiotics colonize the gastrointestinal tract and have the potential to provide many beneficial effects to the host [7]. Recently, several meta-analyses demonstrated that probiotics significantly reduced the risk of NEC and overall mortality in preterm infants [8–12]. Even though breast milk is known to reduce the risk of NEC [13, 14], probiotics turned out to be beneficial also in studies comparing mother’s breast milk with and without supplementation of probiotics [15–18]. Best effects were obtained for multiple-strain probiotics (e.g. Infloran) that contain *Lactobacillus acidophilus* and *Bifidobacterium infantis* [11, 16, 17, 19–23]. However, probiotic treatment of preterm infants is not routine practice in many neonatal departments. Reasons for this are mainly controversial debates about the safety of probiotics, but also uncertainty in the choice of probiotic products, strains and protocols [17]. One safety issue concerns the effect of probiotics on the development of BSI. Three cases of bacteremia with the probiotic species *Bifidobacterium* spp. were described recently in newborns receiving probiotics in a Swiss and a German NICU [24, 25]. Further, a Taiwanese randomized control trial (RCT) including 430 preterm infants reported a higher, but not statistically significant Gram-negative BSI rate in the study group that received probiotics [16]. However, all meta-analyses and systematic reviews recently conducted on this topic reported unchanged [8, 10, 12, 26, 27] or even lower [28] BSI rates after probiotic treatment. Another safety issue refers to the quality of commercially available probiotics. For use in preterm infants only probiotics produced under strict quality control conditions should be recommended. This is the case for probiotic products with licensing as a drug by a regulatory authority such as Infloran [23].

The aim of this study was to assess and evaluate complications of preterm birth (NEC, overall mortality, mortality following NEC and nosocomial BSI) in VLBW infants before and after the implementation of dual-strain probiotics. In addition, a subgroup analysis in ELBW infants was conducted to identify protective and risk factors for complications of preterm births in this special sub cohort.

## Materials and Methods

### Data source

This retrospective multi-center study is based on NEO-KISS, the German surveillance system for nosocomial infections in VLBW infants. Since 2005, all NICUs caring for VLBW infants in Germany participate in this patient-based prospective surveillance system in order to receive reimbursement [5]. Full data collection has already been conducted by German NICUs that voluntarily participated in NEO-KISS since 2000 [5]. In NEO-KISS, surveillance is conducted by trained nurses and doctors who collect demographic data (e.g. birth weight, sex, admission date, gestational age, date of discharge), type of delivery and clinical data (e.g. type of infection, clinical findings, device association) for all VLBW infants. Surveillance by the NEO-KISS database ends, when the infant weighs more than 1,800 g, dies or is transferred to another department.

### Study design and setting

This multi-center time series analysis used NEO-KISS data between 2004 and 2014. In 2011, a survey about routine administration of probiotics was conducted among all German NICUs (n = 229). 168 (73.4%) NICUs responded. All neonatal wards that did not use prophylactic enteral probiotics at all (n = 109), or did not provide sufficient data (n = 11) were excluded from analysis. For validation purposes, the remaining 48 NICUs were contacted by email and/or phone in a second survey in 2014. NICUs that did not respond (n = 1), did not routinely administer probiotics (n = 2) or used probiotic products with a single probiotic species (n = 1) were also excluded from analysis. NICUs were included in the study, if they met the following inclusion criteria: i) routine use of prophylactic enteral probiotics with a multiple-strain product such as Infloran containing *Lactobacillus acidophilus* and *Bifidobacterium* spp. on the neonatal ward level, ii) definition of a start date of implementation, iii) validation of their data in a second survey (2014). 44 NICUs fulfilled the inclusion criteria and provided sufficient data before and after the start of the exposure. A flow chart of included NICUs is depicted in S1 Fig. Characteristics of all NICUs included are shown in S1 Table.

### Probiotic product

All NICUs included in this study used Infloran (Laboratorio Farmaceutico, Mede, Italy), a commercially available combination of *Lactobacillus acidophilus* and *Bifidobacterium infantis*. Infloran is licensed by the Swiss Agency for Therapeutic Products of the Federal Office of Public Health in Switzerland (#00679), SwissMedic, as a drug for use in infants with diarrhea [23]. In consequence, this dual-strain probiotic product is available in drug quality.

### Patients

For the statistical analyses, all preterm infants from the 44 departments included that were admitted between 36 months before and 36 months after the start of exposure (the start date of routine administration of dual-strain probiotics) were considered for analysis. Infants with admission before and discharge after the start of exposure and infants within the first 30 days of the start of exposure (wash-in phase) were excluded. Additionally, infants with missing values in patient based confounding parameters were excluded.

### Primary and secondary outcomes

The primary outcome of this study was NEC until achieving 1800 g, transfer from NICU or death. Secondary outcomes were overall mortality, mortality following NEC and nosocomial primary BSI. Nosocomial BSI was defined as BSI acquired in hospital after the first 72 h of life or 72 h after admission. Criteria for the diagnosis of NEC and BSI were recently described by the European Center for Disease Prevention and Control (ECDC) [29]. The NEO-KISS protocol with definitions of NEC and BSI can be found at http://www.nrz-hygiene.de/fileadmin/nrz/module/neo/NEO-KISSProtocol_english_240210.pdf.

### NEC

For the diagnosis of NEC a combination of one radiological sign (pneumoperitoneum; pneumatosis intestinalis; unchanged rigid loops of small intestine) and two clinical symptoms (vomiting, abdominal distention, persistent microscopic or gross blood in stools, redness of *regio abdominalis lateralis* (flanks) and prefeeding residuals) is required. Alternatively, a documentation of histological diagnosis based on prepared specimens was judged as a criterion for NEC. Histological diagnosis of NEC was taken as proof of NEC and evidence for the distinction from spontaneous perforation of intestine (SPI) [29].

According to the literature, radiographic signs are known to have a high specificity and a low sensitivity [30, 31]. “Fixed loops of the small intestine” was defined as good indication for operation in NEC with a prevalence of 8.5%, a sensitivity of 12.5% and a specificity of 100% [32]. Coursey and colleagues reported “rigid / fixed bowel loops” as an indicator of severity of illness in neonates with NEC. They found this symptom in 10 of 43 (23.3%) infants with suspected NEC, who underwent surgery and in 0 of 86 infants with suspected NEC without surgery [30].

Mortality following NEC was defined by death chronologically after the diagnosis of NEC until end of surveillance.

In NEO-KISS, histological diagnosis of NEC can be documented voluntarily. A histologic specimen was obtained during surgery and could be an indicator for severe cases of NEC [33]. NEC was stratified by NEC type (No NEC, surgical NEC, medical NEC, NEC type unknown) to account for severity. Surgical NEC was defined as NEC with histological diagnosis (after surgery), medical NEC was defined as NEC clinically diagnosed with information that no histological specimen was obtained. NEC type unknown was defined as clinically diagnosed without information on histology. The category no NEC included preterm infants without diagnosis of NEC. Surgery was assumed to be an indicator of severe cases of NEC [33].

### BSI

The cases of primary BSI were stratified in clinically-diagnosed BSI and laboratory-confirmed diagnosis of BSI. The latter was further classified by proven pathogens in two groups, coagulase negative staphylococci only (CoNS) or other than CoNS [29]. CVC- and PVC-associated BSI were defined as a BSI with CVC or PVC present at the onset of the infection.

#### Clinically-diagnosed BSI

For the definition of clinically-diagnosed BSI all of the following criteria must be met:

- Treating physician instituted appropriate antimicrobial therapy for BSI for at least 5 days. A therapy day was similar to an antibiotic day in that it was a “day on which a patient received systematic antibiotic (oral or parenteral)”. The day on which the first dosage was given was counted as the first therapy day, and the day on which the last dosage was given was counted as the last therapy day. This was independent of the number of dosages, their presumed effectiveness or the duration of their effects.
- No pathogens detected in blood culture or blood cultures were not performed. One-time evidence of CoNS in blood culture could not exclude the diagnosis of clinical BSI. Clinical BSI could also be diagnosed under the following conditions: i) CoNS appeared once in blood culture, but could be considered contamination of the blood culture and ii) remaining criteria for CoNS BSI were not fulfilled, but the criteria for clinical BSI were fulfilled.
- No apparent infection at another site.
- In addition, two of the following criteria must be met (without other recognized cause): fever > 38°C, hypothermia < 36.5°C or temperature instability, tachycardia (> 200 / min) or new / more frequent bradycardia (< 80 / min), new or more frequent apnoea (> 20 sec), extended recapillarization time (> 2 sec), unexplained metabolic acidosis (BE < -10 mEq/l), new hyperglycaemia (> 140 mg/dl), other signs of BSI such as skin color (when recapillarization time is not used), laboratory evidence (C-reactive protein, interleukin), increased O_2_ requirement (intubation), unstable condition, apathy. Interleukin must be used as a parameter when laboratory specifications for a pathological value were fulfilled. Interleukin 6–8 was considered.

#### Laboratory-confirmed BSI

Laboratory-confirmed BSI with pathogens other than CoNS required the following criteria:

- A recognized pathogen other than CoNS cultured from blood or cerebrospinal fluid. The latter was included because meningitis in VLBW infants is usually haematogenous. Thus, positive cerebrospinal fluid could be regarded as evidence of BSI even if blood culture were negative or not taken. The pathogen must not be related to infections at other sites.
- In addition, at least two of these symptoms must be present: fever > 38°C, hypothermia < 36.5°C or temperature instability, tachycardia (> 200 / min) or new / more frequent bradycardia (< 80 / min), new or more frequent apnoea (> 20 sec), extended recapillarization time (> 2 sec), unexplained metabolic acidosis (BE < -10 mEq/l), new hyperglycaemia (> 140 mg/dl), other signs of BSI such as skin color (when recapillarization time is not used), laboratory evidence (C-reactive protein, interleukin), increased O_2_ requirement (intubation), unstable condition, apathy. Interleukin must be used as a parameter when laboratory specifications for a pathological value were fulfilled. Interleukin 6–8 was considered.

The definition laboratory-confirmed BSI with CoNS required the following criteria:

- Presence of CoNS in blood or isolated from catheter tip as sole pathogen
- And one of the following laboratory parameters (without another recognized cause) had to be fulfilled: thrombocytes < 100 / nl, ratio between immature granulocytes and total granulocytes > 0.2, leukocytes < 5 / nl (without erythroblasts), C-reactive protein > 2.0 mg / ml or interleukin.
- In addition two of the following criteria (without another recognized cause) needed to be fulfilled: fever > 38°C, hypothermia < 36.5°C or temperature instability, tachycardia (> 200 / min) or new / more frequent bradycardia (< 80 / min), new or more frequent apnoea (> 20 sec), extended recapillarization time (> 2 sec), unexplained metabolic acidosis (BE < -10 mEq/l), new hyperglycaemia (> 140 mg/dl), other signs of BSI such as skin color (when recapillarization time is not used), laboratory evidence (C-reactive protein, interleukin), increased O_2_ requirement (intubation), unstable condition, apathy. Interleukin must be used as a parameter when laboratory specifications for a pathological value were fulfilled. Interleukin 6–8 was considered.

### Patient and NICU associated risk factors

The following patient associated risk factors and confounders were considered in the analyses: birth weight in 250 g steps (< 500 g, 500–749 g, 750–999 g, 1000–1249 g, 1250–1499 g), gestational age defined as completed week of pregnancy (< 27, 27–28, 29–30, > 30 weeks), sex (male/ female), mode of delivery (planned Caesarian section, emergency Caesarian section, vaginal delivery), birth location (inhouse, immediate postnatal transport defined by admission ≤ 72 h after birth, longterm postnatal transport defined by admission > 72 h after birth, missing) and pneumonia. Criteria for the diagnosis of pneumonia can be found in the NEO-KISS protocol (http://www.nrz-hygiene.de/fileadmin/nrz/module/neo/NEO-KISSProtocol_english_240210.pdf) and were recently described by the ECDC [29]. Briefly, one radiological finding (new or progressive infiltrate, shadowing, fluid in the interpleural cavity or interlobar fissure) in combination with deterioration in oxygenation and at least four other clinical findings (temperature > 38°C or < 36.5°C or temperature instability, tachycardia or bradycardia, tachypnoea or apnoea, dyspnoea, increased respiratory secretions, new onset of purulent sputum, isolation of a pathogen from respiratory secretions, C-reactive protein > 2.0 mg/dL, I/T ratio > 0.2) were required to diagnose pneumonia. Patients with severe infections suffer from BSI and / or pneumonia.

The covariable small for gestational age (SGA) was defined as birth weight < 10% percentile, appropriate for gestational age (AGA) as 10–90% percentile, and large for gestational age (LGA) as > 90% percentile based on population-based percentiles, separately for male and female infants as well as for singletons and twins. Twin percentiles were used for all multiple births.

In addition, the following NICU associated risk factors and confounders were used for the analyses: level of center (level I or II perinatal center, obstetrical hospital), type of hospital (university hospital, other teaching hospital, others), size of department (< 20 or ≥ 20 beds), annual number of admission in 2010 (< 30, 30–59, ≥ 60 VLBW infants) and year (as indicator of improvement in neonatal care). Only infants with complete information about all relevant risk factors were included in the analyses.

### Statistical methods

Time series analysis was conducted for patients admitted between 36 months before and 36 months after routine administration of dual-strain probiotics. Half-yearly incidences of primary outcomes were chosen to visualize potential fluctuations of the incidences. In the descriptive analyses percent or median and interquartile range (25% and 75% percentile) were calculated. Differences were tested by Chi-square test. P-values less than 0.05 were considered significant.

Interrupted time series analysis was used to evaluate longitudinal effects of routine probiotic medication on the frequency of NEC, overall mortality, mortality following NEC and nosocomial BSI [34].

Cox-proportional hazard regression was performed in the multivariable analysis to calculate adjusted hazard-ratios (HR) with 95% confidence intervals (95% CI) and evaluate the effect of probiotics. All confounding parameters were parameterized as continuous or dummy parameters and added one degree of freedom to the model. The multivariable model building strategy was performed in a stepwise approach. The selection criterion for including parameter in the model was p ≤ 0.05 and for excluding p ≥ 0.06. P-values less than 0.05 were considered significant. All analyses were performed using SPSS (IBM SPSS statistics, Somer, NY, USA) and SAS (SAS Institute, Cary, NC, USA).

### Ethics / data security

The purpose of this study was to improve quality of neonatal care by analyzing anonymous unit-based data collected by hospitals in accordance with the German “Protection against Infection Act” [35]. Therefore ethical approval and informed consent were not required and institutional review boards were not consulted.

## Results

**Fig 1:**
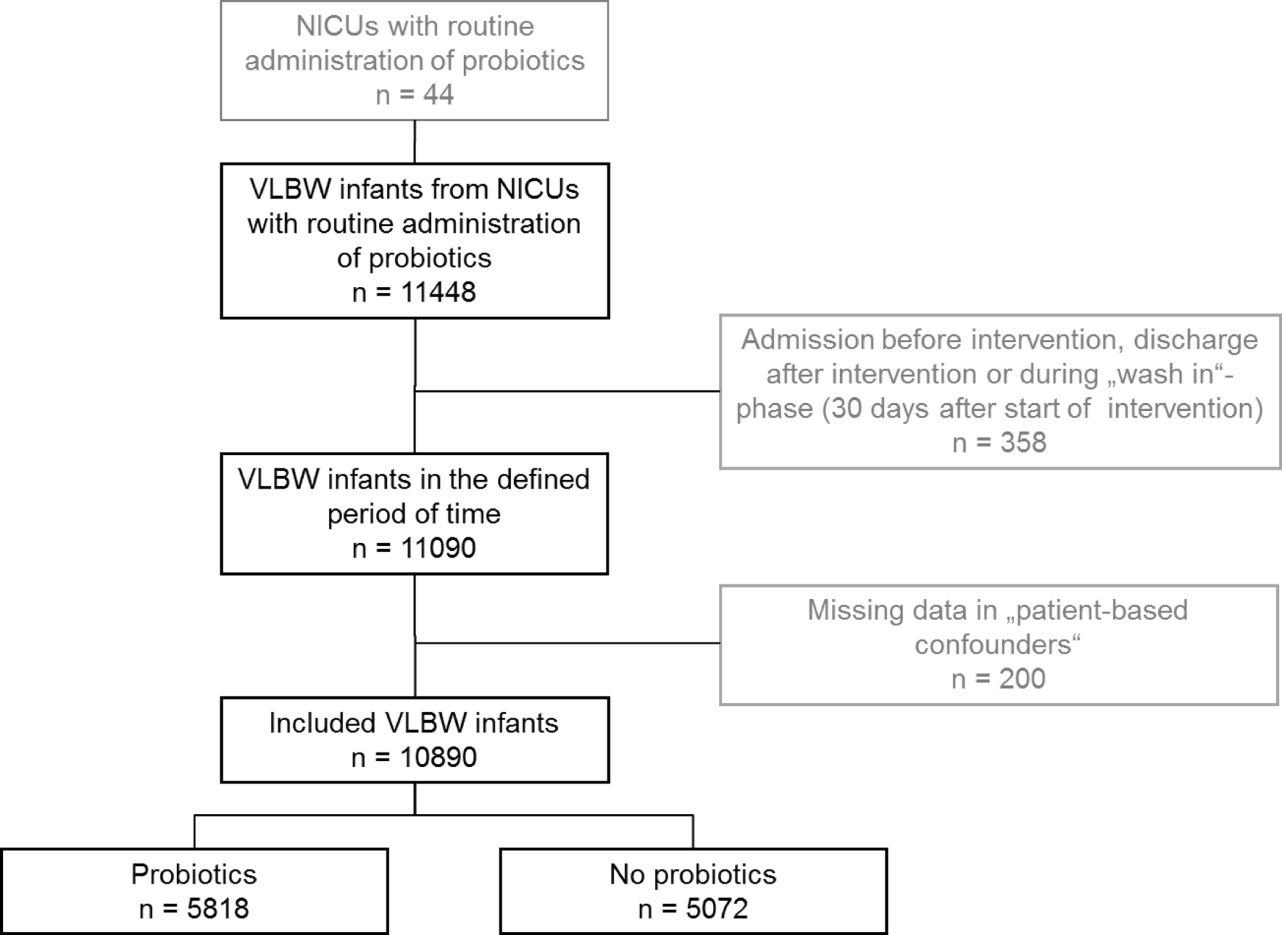
Flow chart of VLBW infants eligible for this study.

The intervention was introduced in July 2010 (January 2010–September 2010) [Median (IQR)]. In the 44 departments, 11,448 infants were admitted 36 months before or after the start date of intervention. 358 infants that were admitted before and discharged after the start of intervention or admitted between the start of intervention and 30 days after the start (“wash in”-phase) were excluded from analysis. An additional 200 infants were excluded due to missing values in patient based confounding parameters. 10,890 infants were included in the analysis. A flow chart summarizing the VLBW infants eligible for this study is depicted in Fig 1. The descriptive analysis of all 10,890 VLBW infants included, stratified by routine use of probiotics, is documented in Table 1.

**Table 1:** Descriptive characteristics of 10,890 VLBW infants included in the study (stratified by routine use of probiotics).

	No probiotics	Probiotics	
Parameter	Number (%) or median (IQR)	Number (%) or median (IQR)	P-value
**Patients**	5072 (100.0%)	5818 (100.0%)	
**Birth weight [250g steps]**			
< 500 g	151 (3.0%)	249 (4.3%)	<0.001*
500–749 g	808 (15.9%)	961 (16.5%)	
750–999 g	1178 (23.2%)	1336 (23.0%)	
1000–1249 g	1132 (22.3%)	1402 (24.1%)	
1250–1499 g	1803 (35.5%)	1870 (32.1%)	
**Gestational age [days]**	203 (188–217)	202 (187–216)	
**Gestational age [group]**			
< 27 weeks	1282 (25.3%)	1599 (27.5%)	0.013*
27–28 weeks	1167 (23.0%)	1387 (23.8%)	
29–30 weeks	1304 (25.7%)	1411 (24.3%)	
> 30 weeks	1319 (26.0%)	1421 (24.4%)	
**Female sex**	2451 (48.3%)	2888 (49.6%)	0.171
**Delivery mode**			
Caesarean section	4216 (83.1%)	4889 (84.0%)	0.008*
Emergency Caesarean section	337 (6.6%)	428 (7.4%)	
Vaginal	517 (10.2%)	501 (8.6%)	
Missing	2 (0.0%)	0 (0.0%)	
**Multiple birth**	1577 (31.1%)	1895 (32.6%)	0.099
**CRIB Score**	2 (1–6)	2 (1–5)	
**Surveillance end point**			
Over 1800 g	4276 (84.3%)	4961 (85.3%)	0.065
Transfer	463 (9.1%)	521 (9.0%)	
Death	329 (6.5%)	336 (5.8%)	
Missing	4 (0.1%)	0 (0.0%)	
**Died**	329 (6.5%)	336 (5.8%)	0.122
**Birth location**			
Inhouse birth	4662 (91.9%)	5506 (94.6%)	<0.001*
Immediate postnatal transport	177 (3.5%)	159 (2.7%)	
Longterm postnatal transport	93 (1.8%)	150 (2.6%)	
Missing	140 (2.8%)	3 (0.1%)	
**NICU days**	33 (23–50)	33 (22–49)	
**NICU days [group]**			
< 21	947 (18.7%)	1223 (21.0%)	0.007*
21–34	1700 (33.5%)	1858 (31.9%)	
35–48	1049 (20.7%)	1240 (21.3%)	
> 48	1376 (27.1%)	1497 (25.7%)	
**CVC days**	6 (0–13)	6 (0–13)	
**PVC days**	8 (3–14)	7 (2–12)	
**ETT days**	0 (0–5)	0 (0–4)	
**CPAP days**	5 (1–20)	8 (2–26)	
**Antibiotic days**	7 (3–14)	6 (2–12)	
**CVC use**	2998 (59.1%)	3443 (59.2%)	0.941
**PVC use**	4446 (87.7%)	5019 (86.3%)	0.032*
**VC use**	4996 (98.5%)	5659 (97.3%)	<0.001*
**ETT use**	2379 (46.9%)	2622 (45.1%)	0.055
**CPAP use**	4004 (78.9%)	4823 (82.9%)	<0.001*
**Respiratory support**	4353 (85.8%)	5151 (88.5%)	<0.001*
**Antibiotic use**	4090 (80.6%)	4485 (77.1%)	<0.001*
**Severe infection (BSI and/or pneumonia)**	904 (17.8%)	951 (16.3%)	0.041*
**Pneumonia**	155 (3.1%)	142 (2.4%)	0.049*
**BSI**	785 (15.5%)	846 (14.5%)	0.172
**CVC-associated BSI**	357 (7.0%)	363 (6.2%)	0.094
**PVC-associated BSI**	348 (6.9%)	349 (6.0%)	0.067
**CVC- and PVC-associated BSI**	680 (13.4%)	694 (11.9%)	0.020*
**NEC**	174 (3.4%)	100 (1.7%)	<0.001*
**NEC type**			
No NEC	4898 (96.6%)	5718 (98.3%)	<0.001*
Surgical NEC	73 (1.4%)	54 (0.9%)	
Medical NEC	56 (1.1%)	22 (0.4%)	
NEC type unknown	45 (0.9%)	24 (0.4%)	
**Time to first NEC [days]**	18 (10–29)	15 (10–24)	
**Time from first NEC to end of surveillance [days]**	22 (4–49)	36 (9–61)	
**Time to first NEC or discharge**	33 (23–49)	32 (22–48)	
**Birth year**			
2004	25 (0.5%)	0 (0.0%)	<0.001*
2005	27 (0.5%)	0 (0.0%)	
2006	242 (4.8%)	18 (0.3%)	
2007	917 (18.1%)	30 (0.5%)	
2008	1646 (32.5%)	73 (1.3%)	
2009	1474 (29.1%)	398 (6.8%)	
2010	687 (13.5%)	1161 (20.0%)	
2011	52 (1.0%)	1720 (29.6%)	
2012	2 (0.0%)	1550 (26.6%)	
2013	0 (0.0%)	811 (13.9%)	
2014	0 (0.0%)	57 (1.0%)	
**Size of unit [beds]**			
< 20	1298 (25.6%)	1674 (28.8%)	<0.001*
≥ 20	3774 (74.4%)	4144 (71.2%)	
**Size of hospital [beds]**			
< 600	1972 (38.9%)	2267 (39.0%)	0.928
≥ 600	3100 (61.1%)	3551 (61.0%)	
**Neonatal care level**			
Perinatal center level I	5011 (98.8%)	5719 (98.3%)	0.067
Perinatal center level II	45 (0.9%)	79 (1.4%)	
Obstetric clinic	16 (0.3%)	20 (0.3%)	
**Type of hospital**			
University hospital	1870 (36.9%)	2125 (36.5%)	0.542
Other teaching hospital	2853 (56.3%)	3261 (56.1%)	
Other hospital	349 (6.9%)	432 (7.4%)	
**Growth**			
AGA	3434 (67.7%)	3992 (68.6%)	0.015*
SGA	1463 (28.8%)	1638 (28.2%)	
LGA	164 (3.2%)	157 (2.7%)	
Missing	11 (0.2%)	31 (0.5%)	

AGA–Appropriate for gestational age, BSI- blood stream infection, CPAP–Continuous nasal positive airway pressure, CRIB–Clinical risk index for babies, CVC–Central venous catheter, ETT–Endotracheal tube, IQR–interquartile range, LGA–Large for gestational age, Patient days–Total days present on department, PVC–Peripheral venous catheter, Respiratory support includes CPAP and ETT, SGA–Small for gestational age, VC–Venous catheter. Chi-square statistics were performed for categorical variables.

* P-values < 0.05 were interpreted as significant.

S2 Table depicts the descriptive analysis of all 4,683 ELBW infants included, stratified by routine use of probiotics.

### NEC

**Fig 2:**
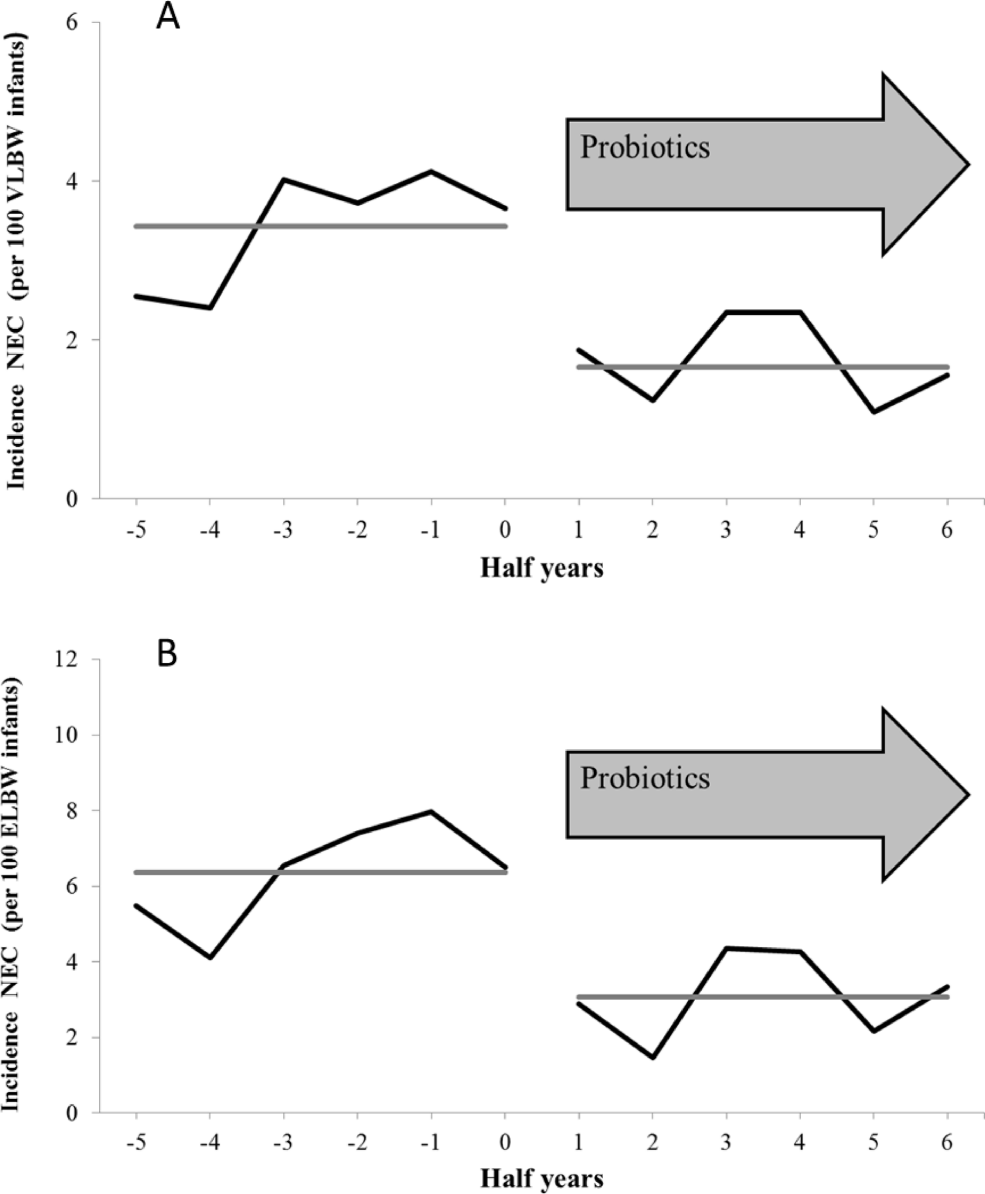
**NEC in VLBW infants (A) and in ELBW infants (B) treated in 44 neonatal departments before and after the routine medication of probiotics. **Half-yearly incidences of NEC in 10,890 VLBW infants (A) and in 4,683 ELBW infants (B) treated in 44 neonatal departments before and after the routine medication of probiotics. The grey line represents the trend of NEC incidences (per 100 VLBW/ELBW infants) before and after the introduction of routine administration of probiotics.

Of the 10,890 VLBW infants eligible for this study, 2.5% (n = 274) suffered from NEC. 4.6% of 4,683 ELBW infants (n = 215) developed NEC during the study period. The half-yearly incidences of NEC (per 100 VLBW or ELBW infants) decreased with routine use of dual-strain probiotics (Fig 2A and 2B). The Cox proportional hazard regression identified routine probiotic treatment to be protective against NEC in VLBW and ELBW infants (Table 2). Further independent risk and protective factors for NEC in VLBW and ELBW infants are summarized in Table 2.

**Table 2:** Cox-proportional-hazard regression model with the outcome NEC in VLBW and ELBW infants.

	VLBW		ELBW	
Parameter	HR	95% CI;p-value	HR	95% CI;p-value
**Probiotics**	0.484	0.378–0.619; p < 0.001	0.481	0.364–0.635; p < 0.001
**Birth weight < 500 g**	3.969	2.277–6.918; p < 0.001	2.184	1.355–3.521; p = 0.0013
**Birth weight 500–749 g**	3.723	2.463–5.628; p < 0.001	2.016	1.477–2.752; p < 0.001
**Birth weight 750–999 g**	1.903	1.301–2.783; p < 0.001		
**Gestational age (26 weeks and younger)**	1.812	1.312–2.502; p < 0.001	1.723	1.236–2.402; p = 0.0013
**Large for gestational age (LGA)**	1.995	1.157–3.438; p = 0.013	2.315	1.235–4.340; p = 0.009
**≥ 60 VLBWs per year**	0.625	0.478–0.817; p < 0.001	0.681	0.508–0.912; p = 0.01
**Immediate postnatal transport**	1.938	1.129–3.328; p = 0.016	2.508	1.425–4.414; p = 0.014

Results of multivariable analysis: segmented regression analysis of interrupted time series using a Cox-proportional-hazard regression model with the outcome NEC in 10,890 VLBW infants and 4,683 ELBW infants (<1000 g) in the time period 3 years before and 3 years since administration of routinely use of probiotics. 95% CI–95% confidence interval.

### Mortality

**Fig 3:**
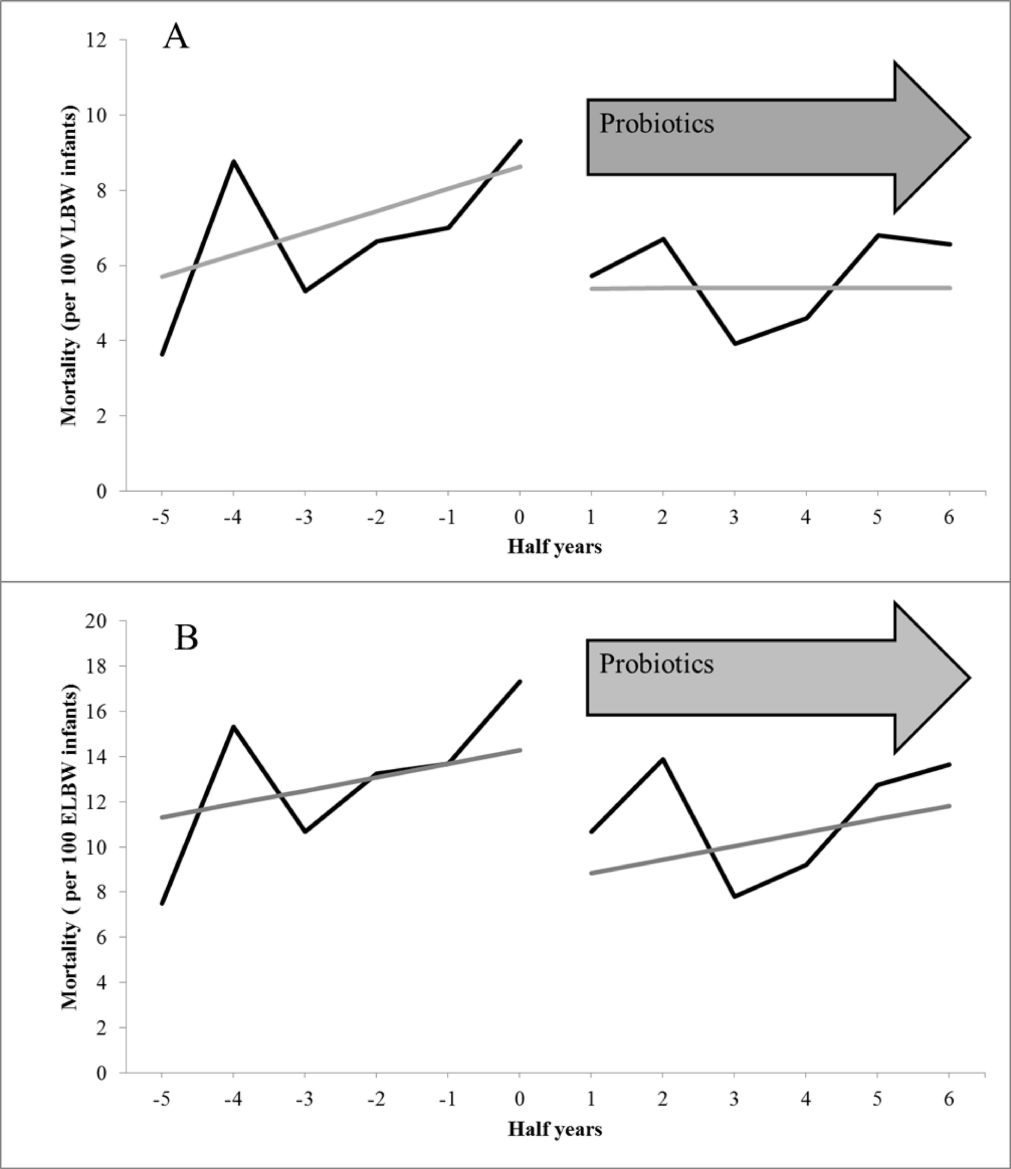
**Overall mortality in VLBW infants (A) and in ELBW infants (B) before and after the routine medication of probiotics. **Half-yearly overall mortality in 10,890 VLBW infants (A) and in 4,683 ELBW infants (B) treated in 44 neonatal departments before and after the routine medication of probiotics. The grey line represents the trend of mortality (per 100 VLBW/ELBW infants) before and after the introduction of routine administration of probiotics.

665 of 10,890 VLBW infants (6.1%) died during the study period. Mortality rate was 11.9% among ELBW infants (557 of 4,683). The half-yearly overall mortality rates (per 100 VLBW or ELBW infants) are shown in Fig 3. Probiotics were associated with lower mortality in VLBW and ELBW infants (Table 3). Further independent risk factors identified by Cox proportional hazard regression for VLBW and ELBW infants are summarized in Table 3.

**Table 3:** Cox-proportional-hazard regression model with the outcome overall mortality VLBW and ELBW-infants.

	VLBW		ELBW	
Parameter	HR	95% CI; p-value	HR	95% CI; p-value
**Time trend before probiotics (per month)**	1.018	1.006–1.030; p = 0.002	1.009	1.001–1.018; p = 0.036
**Probiotics**	0.604	0.442–0.826; p = 0.002	0.587	0.411–0.837; p = 0.003
**Change in time trend after probiotics (per month)**	0.982	0.967–0.997; p = 0.021		
**Birth weight < 500 g**	10.783	6.958–16.711; p < 0.001	8.353	6.058–11.517; p < 0.001
**Birth weight 500–749 g**	3.871	2.726–5.496; p < 0.001	2.768	2.206–3.473; p < 0.001
**Birth weight 750–999 g**	1.431	1.032–1.985; p = 0.032		
**Gestational age (≤ 26 weeks)**	3.268	2.193–4.869; p < 0.001	1.980	1.493–2.624; p < 0.001
**Gestational age (27 and 28 weeks)**	1.597	1.129–2.259; p = 0.008		
**Male**	1.569	1.340–1.838; p < 0.001	1.617	1.362–1.919; p < 0.001
**Multiple birth**	1.262	1.067–1.492; p = 0.007	1.348	1.126–1.615; p = 0.001
**Vaginal**	1.819	1.482–2.234; p < 0.001	2.081	1.681–2.577; p < 0.001
**Emergency Caesarean section**	1.647	1.290–2.105; p < 0.001	1.713	1.315–2.232; p < 0.001
**Small for gestational age (SGA)**	0.715	0.564–0.905; p = 0.005	0.603	0.465–0.781; p < 0.001
**Large for gestational age (LGA)**	1.581	1.093–2.285; p = 0.015		
**< 30 VLBWs per year**	0.746	0.609–0.915; p = 0.005	0.733	0.585–0.917; p = 0.007
**≥ 60 VLBWs per year**	0.583	0.467–0.729; p < 0.001	0.572	0.448–0.732; p < 0.001
**Immediate postnatal transport**	1.610	1.121–2.312; p = 0.010		

Results of multivariable analysis: segmented regression analysis of interrupted time series using a Cox-proportional-hazard regression model with the outcome overall mortality (without the time dependent variable NEC) in 10,890 VLBW-infants (665 deceased) and in 4,683 ELBW-infants (557 deceased). 95% CI–95% confidence interval.

### Mortality following NEC

44 of the 274 VLBW infants (16.1%) suffering from NEC died. Median time from diagnosis of NEC to death was 6 days (IQR 2–15 days). In the ELBW cohort 39 of the 215 infants suffering from NEC (18.1%) died. The half-yearly mortality rates (per 100 VLBW or ELBW infants with NEC) decreased after routine use of probiotics (S2A and S2B Fig). The multivariable analyses identified that probiotics improved survival of VLBW and ELBW infants suffering from NEC (Table 4). Independent risk factors for mortality following NEC in VLBW and ELBW infants are shown in Table 4. The cumulative survival function for mortality following NEC demonstrated that especially within the first days of NEC probiotics seemed to be beneficial for survival of preterm infants (S3A and S3B Fig).

**Table 4:** Cox-proportional hazard regression with the outcome mortality following NEC in VLBW and in ELBW-infants.

	VLBW		ELBW	
Parameter	HR	95%CI; p-value	HR	95%CI; p-value
**Probiotics**	0.510	0.260–0.999; p = 0.0497	0.397	0.186–0.847; p = 0.017
**Birth weight < 500 g**	3.091	1.555–6.145; p = 0.001	3.105	1.538–6.270; p = 0.002
**Vaginal delivery**	2.129	1.048–4.326; p = 0.037	2.219	1.076–4.574; p = 0.031

Results of multivariable analysis: interrupted time series with segmented regression using Cox-proportional hazard regression with the outcome mortality following NEC in 274 VLBW infants (44 deceased) and in 215 ELBW infants (39 deceased). 95% CI–95% confidence interval.

### BSI

**Fig 4:**
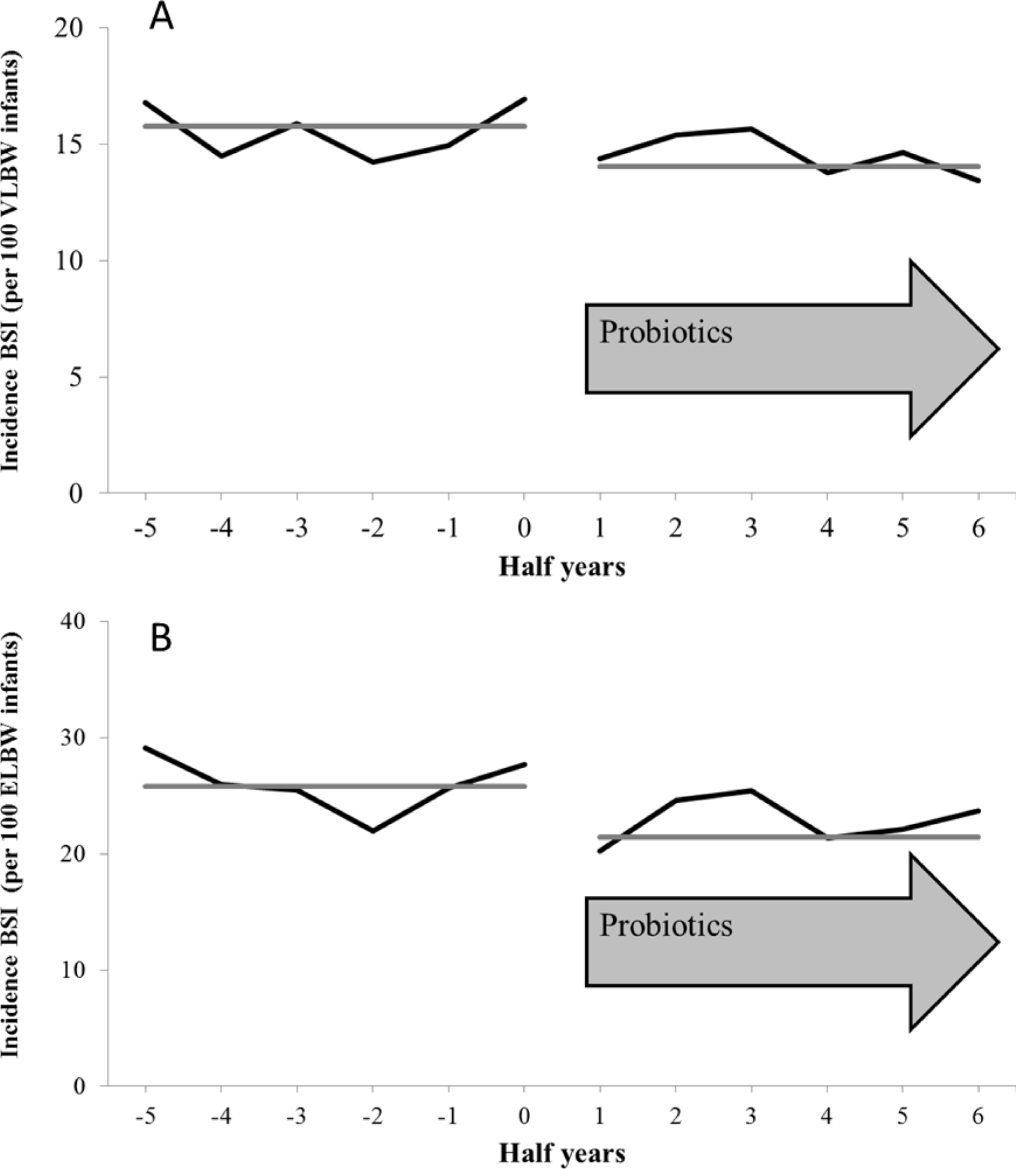
**Nosocomial BSI in VLBW (A) and in ELBW infants (B) before and after the routine medication of probiotics. **Half-yearly incidences of nosocomial BSI in 10,890 VLBW (A) and in 4,683 ELBW infants (B) treated in 44 neonatal departments before and after the routine medication of probiotics. The grey line represents the trend of incidences of bloodstream infections (per 100 VLBW/ELBW infants) before and after the introduction of routine administration of probiotics.

1,631 of 10,890 VLBW infants (15.0%) suffered from nosocomial BSI during the study period. 24.2% of 4,683 ELBW infants (n = 1,133) developed nosocomial BSI. 851 of 1,631 BSI were clinically-diagnosed, 385 were laboratory-confirmed with proof of pathogen other than CoNS and 395 BSI were laboratory-confirmed with CoNS as sole pathogen. A decrease of half-yearly incidences of nosocomial BSI among 10,890 VLBW and 4,683 ELBW infants is shown in Fig 4A and 4B. The multivariable analysis suggested that probiotics were associated with lower nosocomial BSI rates in VLBW and ELBW infants (Table 5). Low birth weight, young gestational age and male gender were identified as risk factors for nosocomial BSI in VLBW and ELBW infants (Table 5).

**Table 5:** Cox-proportional-hazard regression model with the outcome nosocomial BSI in VLBW and in ELBW infants.

	VLBW		ELBW	
Parameter	HR	95% CI; p-value	HR	95% CI; p-value
**Probiotics**	0.890	0.807–0.981; p = 0.019	0.832	0.741–0.936; p = 0.002
**Birth weight < 500 g**	2.746	2.192–3.441; p < 0.001	2.059	1.692–2.507; p < 0.001
**Birth weight 500–749 g**	1.976	1.668–2.341; p < 0.001	1.473	1.290–1.680; p < 0.001
**Birth weight 750–999 g**	1.344	1.156–1.563; p < 0.001		
**Gestational age (≤ 26 weeks)**	2.349	1.886–2.925; p < 0.001	2.016	1.592–2.553; p < 0.001
**Gestational age (27 and 28 weeks)**	1.849	1.511–2.262; p < 0.001	1.598	1.247–2.047; p < 0.001
**Gestational age (29 and 30 weeks)**	1.334	1.087–1.636; p = 0.006		
**Male sex**	1.242	1.126–1.371;p < 0.001	1.243	1.106–1.398; p < 0.001

Results of multivariable analysis: segmented regression analysis of interrupted time series using a Cox-proportional-hazard regression model with the outcome nosocomial BSI in 10,890 VLBW infants and in 4,683 ELBW infants in the time period 3 years before and 3 years since introduction of routinely use of probiotics. 95% CI–95% confidence interval.

## Discussion

This large observational multi-center study demonstrated that routine medication with dual-strain probiotics in German neonatal wards was significantly associated with reduced incidences of NEC and overall mortality. These beneficial effects of probiotics were already demonstrated by several meta-analyses and systematic reviews including RCTs [8, 10, 11, 26–28, 36] and observational studies [9], but have never been verified on such a large clinical scale. Recently, Härtel et al. confirmed the association of probiotics with a reduced risk of NEC surgery (OR 0.58, 95% CI, 0.37–0.91) in an observational study including 2,828 VLBW infants in German NICUs [22]. Olsen and colleagues conducted a meta-analysis of 12 observational studies addressing the use of prophylactic probiotics for preterm infants [9]. 3 of these studies also used Infloran with *Lactobacillus acidophilus* and *Bifidobacterium infantis* as probiotic agents [19, 21, 22]. Two studies reported significant reduction of NEC by Infloran [19, 22]; one reported the protective effect of Infloran in the subgroup of preterm infants fed with breast milk [21]. Another recent retrospective cohort study demonstrated the protective effect of Infloran on NEC in two German NICUs and one Swiss NICU [20]. Our study verified the existing data and showed for the first time that probiotic treatment also improved survival of preterm infants already suffering from NEC. This might be due to a milder course of disease facilitated by probiotics and provides important information to improve the outcome of these critically ill patients.

Critics of probiotic use are primarily worried about safety issues. Probiotics are living microorganisms and have the potential to cause infections, predominantly in preterm infants with a premature immune system [37]. Our data supported the findings of recent meta-analyses and systematic reviews that probiotics did not increase BSI rates [8–10, 12, 26–28]. In fact, we showed that probiotic treatment was even protective against nosocomial BSI. One reason, why this effect was not seen by other studies might be their smaller sample sizes. The beneficial effects of probiotics were not only present in VLBW infants, but were even more pronounced in the sub cohort of ELBW infants. Consequently, probiotics with licensing as a drug by a regulatory authority such as Infloran seem to be safe and beneficial even in this vulnerable population.

This study is based on anonymized surveillance data. Thus, we have no additional information on safety monitoring practice by neonatal units regarding probiotic bacteremia. However, the surveillance data showed that 32 (3.8%, CI95% 2.65–5.28%) of 846 VLBW infants who developed BSI and received probiotics died. Mortality rate was 6.2% (n = 49, CI 95% 4.7–8.1%) in the subgroup of VLBW infants who developed BSI and did not receive probiotics (n = 785). Thus, probiotics reduced mortality in the sub cohort of VLBW infants with BSI (RR = 0.61, CI95% = 0.39–0.93). However, for 16 VLBW infants (15 clinically diagnosed BSI, 1 laboratory-confirmed BSI with “other bacteria” as causative agent) we could not exclude a probiotic strain as causative agent of the BSI. No adverse events or cases of bacteremia with probiotic species were reported in studies examining the effect of Infloran [19–22]. Even though probiotic bacteremia might be underestimated due to anaerobic culture conditions required by *Bifidobacterium* spp. this seems to be an extremely rare event [24]. Three cases of bacteremia with *Bifidobacterium* spp. in preterm infants who received Infloran are known in literature [24, 25]. All preterm infants recovered. Thus, in addition to the beneficial effects facilitated by probiotics in preterm infants neonatologists should be aware of the potential of probiotic species to cause infections.

The mechanisms by which probiotics work and might prevent preterm complications remain unclear. Abnormal patterns of microbiota combined with a novel pathogen most likely contribute to the etiology of NEC [38]. Investigations applying 16S rRNA sequencing revealed that the composition of the gut microbiota in preterm infants suffering from NEC changed between one week and < 72 hours before diagnosis of NEC [38]. The authors observed a decrease of Firmicutes including the probiotic species *Lactobacillus acidophilus* by 32% [38]. These findings strongly suggest that a healthy gut microbiota established by probiotic treatment prevents complications of preterm infants including NEC and BSI.

Main strengths of our study are the large sample size and the multi-center study design. Data of more than 10,000 VLBW and more than 4,500 ELBW infants allowed us to identify also small effects such as the protective effect of dual-strain probiotics on nosocomial BSI and mortality following NEC. Further, our non-RCT design added data on effectiveness to the large body of literature existing on the efficacy of dual-strain probiotics for preterm complications. This study has limitations due to its observational, non-RCT study design. The anonymous surveillance data used for this study did not provide information on protocols for probiotic supplementation used by each NICU. Recommendations for dosage, frequency and duration of probiotic prophylaxis with Infloran for preterm infants were recently published [20]. In addition, we lack information on major changes in enteral feeding management or neonatal care that could influence the incidence of NEC. However, risk of confounding by these unaccounted factors was reduced by interrupted time series analysis. This statistical method considered the trends before and after the implementation of probiotics as well as the change of the outcome level after the intervention. Further, we adjusted the multivariable analysis for the factor year to consider the potential impact of general improvement of neonatal care on our results. Year was not identified as independent risk or protective factor for NEC, mortality, mortality following NEC and BSI. Thus, it is highly unlikely that the observed reductions of preterm complications after the intervention are a result of general advances in neonatal care only and cannot be attributed to the protective effect of the dual-strain probiotics. Another limitation might be missed cases of NEC due to end of surveillance. Surveillance for a department in NEO-KISS ended, if the VLBW infant weighed more than 1800 g, ii) died or was iii) transferred to another unit. In consequence, NEC would not be counted for infants weighing more than 1800 g. However, the majority of NEC cases occur in preterm infants with birth weights below 1500 g [4] with the most common age of onset of three days [39]. Further, NEC were not documented by NEO-KISS, if a VLBW infant developed NEC after transfer from another unit and this NEC was diagnosed during the first 72 h after admission to the new NICU. In most cases, however, VLBW infants under development of NEC and / or below a weight of 1800 g are not transferred to another unit. If a child was transferred for surgery because of NEC, this case would be counted in the transferring department. The fact that Bell’s staging was not used for diagnosis or classification of NEC in NEO-KISS is another important issue to discuss [29]. Even though, accuracy of Bell’s criteria has been discussed before [40, 41], it is commonly used in neonatal probiotic literature to quantify severity of NEC [10, 27]. Our data did not account for classification of NEC, even though histological diagnosis of NEC might constitute a surrogate parameter. A histologic specimen is an indicator of surgery and in consequence, severe cases of NEC [33]. Based on these assumptions, we included the stratification of NEC type (No NEC, surgical NEC, medical NEC, NEC type unknown) to the analysis. We re-analyzed our multivariable model with the outcomes surgical NEC, medical NEC and NEC type unknown adjusting for the same cofactors as the original model for all NEC cases. Dual-strain probiotics were protective against all types of NEC suggesting that they also prevented severe cases. As we mentioned before, histological diagnosis of NEC is not a mandatory input field in NEO-KISS. Thus, assumptions underlying these analyses were speculative and should not be used for clinical recommendations.

The study design applying interrupted time series analysis required patient data from 36 months before and 36 months after the implementation of probiotics in the NICUs. In consequence, we could not include those German NICUs that implemented routine use of probiotics after the first survey in 2011. In fact, the number of NICUs that implemented routine probiotic use after 2011 is unknown. The most recent survey was conducted by Härtel and colleagues among 46 German NICUs participating in the German Neonatal Network (GNN) in 2012. Even in this very motivated subgroup, only 34 NICUs (74%) reported routine use of probiotics [22]. In consequence, it is very likely that still many neonatal wards do not use routine probiotic medication for their preterm infants.

## Conclusion

This large multi-center study adds data of more than 10,000 VLBW infants to the existing body of evidence that prophylactic enteral administration of dual-strain probiotics significantly reduces the incidences of NEC, overall mortality, mortality following NEC and BSI. If these severe complications of preterm birth are to be reduced noticeably, the use of dual-strain probiotics should be considered in standard neonatal care, especially for ELBW infants.

## Supporting Information

S1 Fig Flow chart of NICUs included in this study. (TIF)

S2 Fig Mortality in VLBW infants and in ELBW infants with NEC before and after the routine administration of probiotics. Half-yearly mortality in 274 VLBW-infants and in 215 ELBW-infants with NEC treated in 44 neonatal departments before and after the routine medication of probiotics. The grey line represents trend of mortality following NEC (per 100 VLBW infants with NEC) before and after the introduction of routine administration of probiotics.
(TIF)

S3 Fig Cumulative survival functions for mortality following NEC. Cumulative survival functions for mortality following NEC for 274 VLBW-infants (A) and for 215 ELBW-infants (B) with and without routine administration of probiotics. P < 0.001 using Log Rank Test (Cox-Mantel).
(TIF)

S1 Table Descriptive analysis of 44 neonatal departments included in the analysis. IQR–interquartile range, NICU–Neonatal intensive care unit.
(DOCX)

S2 Table Descriptive characteristics of 4,683 ELBW infants included in the study (stratified by routine use of probiotics). AGA–Appropriate for gestational age, BSI- blood stream infection, CPAP–Continuous nasal positive airway pressure, CRIB–Clinical risk index for babies, CVC–Central venous catheter, ETT–Endotracheal tube, IQR–interquartile range, LGA–Large for gestational age, Patient days–Total days present on department, PVC–Peripheral venous catheter, Respiratory support includes CPAP and ETT, SGA–Small for gestational age, VC–Venous catheter. Chi-square statistics were performed for categorical variables. * P-values < 0.05 were interpreted as significant.
(DOCX)
