# Supplementary figures and images for: Protective Effect of Dual-Strain Probiotics in Preterm Infants: A Multi-Center Time Series Analysis
Source: PLoS One. 2016 Jun 22;11(6):e0158136. doi: 10.1371/journal.pone.0158136 (PMC4917100; doi:10.1371/journal.pone.0158136)

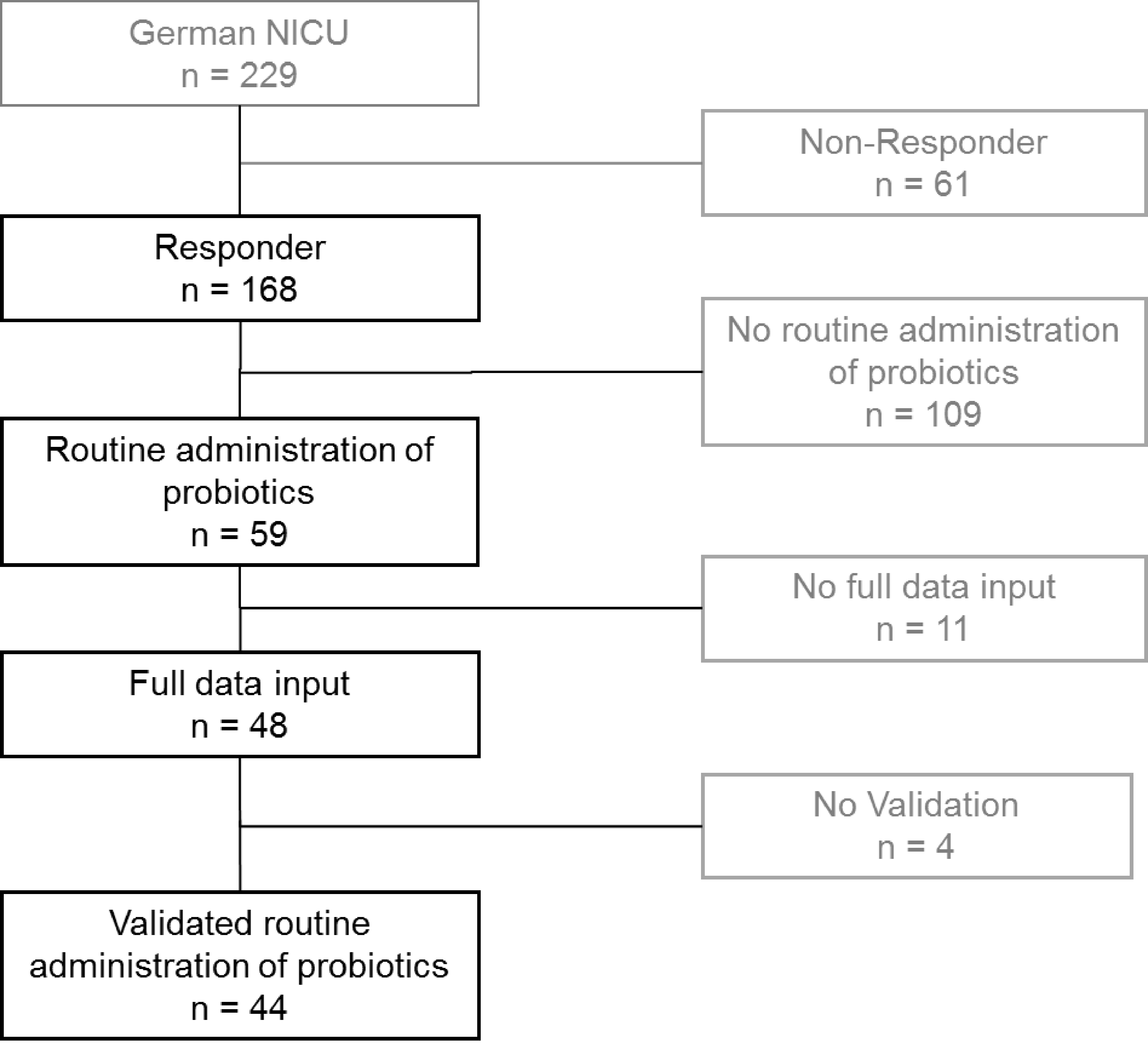

Supplement: S1 Fig — (TIF) [file pone.0158136.s001.tif]

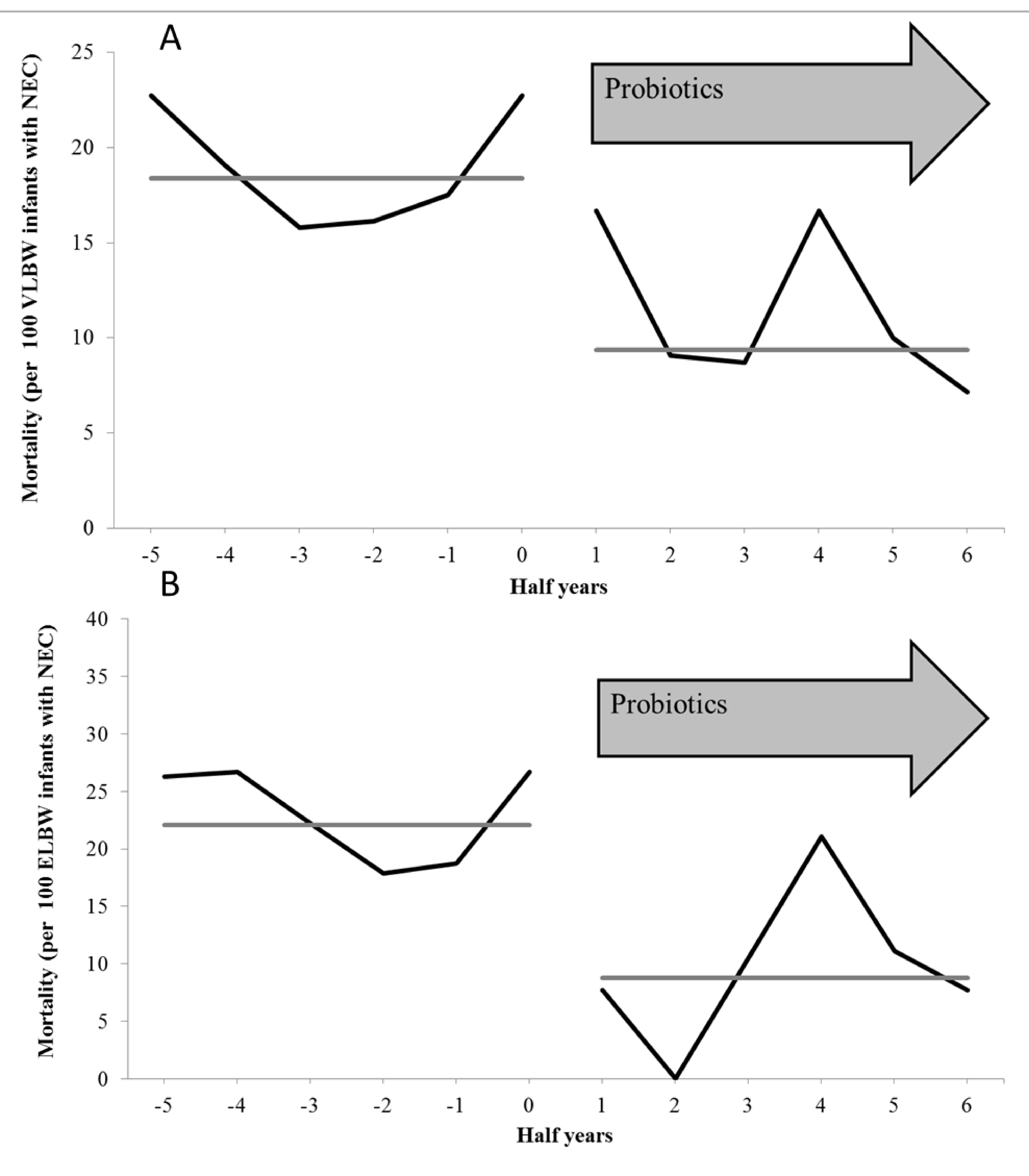

Supplement: S2 Fig — Half-yearly mortality in 274 VLBW-infants and in 215 ELBW-infants with NEC treated in 44 neonatal departments before and after the routine medication of probiotics. The grey line represents trend of mortality following NEC (per 100 VLBW infants with NEC) before and after the introduction of routine administration of probiotics. (TIF) [file pone.0158136.s002.tif]

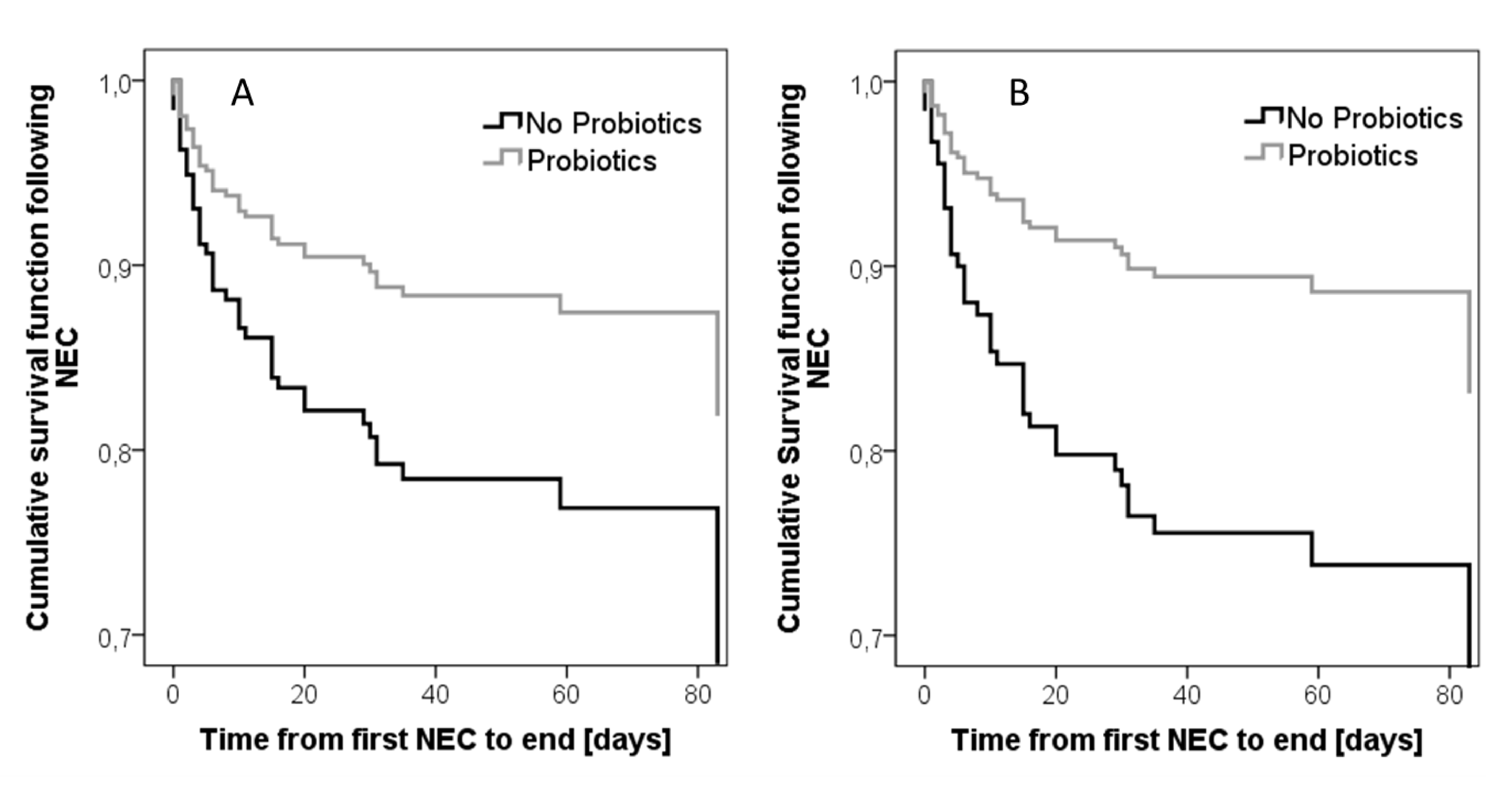

Supplement: S3 Fig — Cumulative survival functions for mortality following NEC for 274 VLBW-infants (A) and for 215 ELBW-infants (B) with and without routine administration of probiotics. P < 0.001 using Log Rank Test (Cox-Mantel). (TIF) [file pone.0158136.s003.tif]
