# Supplementary material for: Protective Effect of Dual-Strain Probiotics in Preterm Infants: A Multi-Center Time Series Analysis
Source: PLoS One. 2016 Jun 22;11(6):e0158136. doi: 10.1371/journal.pone.0158136 (PMC4917100; doi:10.1371/journal.pone.0158136)
Supplement: S1 Table — IQR–interquartile range, NICU–Neonatal intensive care unit. (DOCX) [file pone.0158136.s004.docx]

**Supporting Tables**

S1 Table: Descriptive analysis of 44 neonatal departments included in the analysis.

|  | **Category** | **Number/median** | **[%] /IQR** |
| --- | --- | --- | --- |
| **Neonatal care level** | Perinatal center Level I | 40 | 90.9 % |
|  | Perinatal center Level II | 3 | 6.8 % |
|  | Obstetric clinic | 1 | 2.3 % |
| **Type of hospital** | University hospital | 10 | 22.7 % |
|  | Other teaching hospital | 28 | 63.6 % |
|  | Other hospital | 6 | 13.6 % |
| **Size of unit [number of beds], median, IQR** |  | 21 | 15-29 |
| **Size of hospital [number of beds]** |  | 597 | 468-1067 |
| **Probiotics** |  | 44 | 100.0 % |
| **Administration of probiotics since [date], median, IQR** |  | 14 June 2010 | 1 January 2010 – 1 October 2010 |
| **Infants per unit (total)** |  | 333 | 229-489 |
| **Infants per NICU (before intervention)** |  | 149 | 88-230 |
| **Infants per NICU (after intervention)** |  | 172 | 115-228 |

IQR – interquartile range, NICU – Neonatal intensive care unit.
