# Supplementary material for: Protective Effect of Dual-Strain Probiotics in Preterm Infants: A Multi-Center Time Series Analysis
Source: PLoS One. 2016 Jun 22;11(6):e0158136. doi: 10.1371/journal.pone.0158136 (PMC4917100; doi:10.1371/journal.pone.0158136)
Supplement: S2 Table — AGA–Appropriate for gestational age, BSI- blood stream infection, CPAP–Continuous nasal positive airway pressure, CRIB–Clinical risk index for babies, CVC–Central venous catheter, ETT–Endotracheal tube, IQR–interquartile range, LGA–Large for gestational age, Patient days–Total days present on department, PVC–Peripheral venous catheter, Respiratory support includes CPAP and ETT, SGA–Small for gestational age, VC–Venous catheter. Chi-square statistics were performed for categorical variables. * P-values < 0.05 were interpreted as significant. (DOCX) [file pone.0158136.s005.docx]

**S2 Table: Descriptive characteristics of 4,683 ELBW-infants included in the study (stratified by routine use of probiotics).**

|  | **No probiotics** | **Probiotics** |  |
| --- | --- | --- | --- |
| **Parameter** | **Number (%) or median (IQR)** | **Number (%) or median (IQR)** | **P-value** |
| **Patients** | 2137 (100.0%) | 2546 (100.0%) |  |
| **Birth weight [g]** | 790 (650 – 920) | 780 (640-915) |  |
| **Birth weight [250g steps]** |  |  |  |
| <500 g | 151 (7.1%) | 249 (9.8%) | 0.003* |
| 500-749 g | 808 (37.8%) | 961 (37.7%) |  |
| 750-999 g | 1178 (55.1%) | 1336 (52.5%) |  |
| **Gestational age [days]** | 185 (176-196) | 175 (175-195) |  |
| **Gestational age group** |  |  |  |
| <27 weeks | 1223 (57.2%) | 1519 (59.7%) | 0.137 |
| 27-28 weeks | 596 (27.9%) | 696 (27.3%) |  |
| 20-30 weeks | 237 (11.1%) | 259 (10.2%) |  |
| >30 weeks | 81 (3.8%) | 72 (2.8%) |  |
| **Female sex** | 1043 (48.8%) | 1259 (49.5%) | 0.661 |
| **Delivery mode** |  |  |  |
| Caesarean Section | 1725 (80.7%) | 2087 (82.0%) | 0.131 |
| Emergency Caesarean Section | 166 (7.8%) | 211 (8.3%) |  |
| Missing | 0 (0.0%) | 0 (0.0%) |  |
| Vaginal | 246 (11.5%) | 248 (9.7%) |  |
| **Multiple birth** | 553 (25.9%) | 763 (30.0%) | 0.003* |
| **CRIB Score** | 6 (3-9) | 6 (2-8) |  |
| **Surveillance end point** |  |  |  |
| Over 1800g | 1653 (77.4%) | 1997 (78.4%) | 0.183 |
| Transfer | 211 (9.9%) | 263 (10.3%) |  |
| Death | 271 (12.7%) | 286 (11.2%) |  |
| Missing | 2 (0.1%) | 0 (0.0%) |  |
| **Died** | 271 (12.7%) | 286 (11.2%) | 0.127 |
| **Birth location** |  |  |  |
| Inhouse birth | 1951 (91.3%) | 2393 (94.0%) | <0.001* |
| Immediate postnatal transport | 73 (3.4%) | 63 (2.5%) |  |
| Longterm postnatal transport | 59 (2.8%) | 88 (3.5%) |  |
| Missing | 54 (2.5%) | 2 (0.1%) |  |
| **NICU days** | 54 (40-66) | 51 (38-64) |  |
| **NICU days [group]** |  |  |  |
| <21 | 311 (14.6%) | 354 (13.9%) | 0.001* |
| 21-34 | 111 (5.2%) | 154 (6.0%) |  |
| 35-48 | 423 (19.8%) | 613 (24.1%) |  |
| >48 | 1292 (60.5%) | 1425 (56.0%) |  |
| **CVC days** | 12 (4-22) | 11 (4-19) |  |
| **PVC days** | 9 (2-17) | 7 (1-14) |  |
| **ETT days** | 4 (0-15) | 3 (0-13) |  |
| **CPAP days** | 19 (4-33) | 25 (2-38) |  |
| **Antibiotic days** | 13 (7-24) | 10 (5-10) |  |
| **CVC use** | 1751 (81.9%) | 2080 (81.7%) | 0.832 |
| **PVC use** | 1764 (82.5%) | 2079 (81.7%) | 0.430 |
| **VC use** | 2106 (98.5%) | 2466 (96.9%) | <0.001* |
| **ETT use** | 1529 (71.5%) | 1724 (67.7%) | 0.005* |
| **CPAP use** | 1820 (85.2%) | 2230 (87.6%) | 0.016* |
| **Respiratory support** | 2068 (96.8%) | 2468 (96.9%) | 0.747 |
| **Antibiotic use** | 2007 (93.9%) | 2313 (90.8%) | <0.001* |
| **Severe infection (BSI and /or pneumonia)** | 644 (30.1%) | 668 (26.2%) | 0.003* |
| **Pneumonia** | 126 (5.9%) | 120 (4.7%) | 0.071 |
| **BSI** | 552 (25.8%) | 581 (22.8%) | 0.017* |
| **CVC-associated BSI** | 284 (13.3%) | 286 (11.2%) | 0.032* |
| **PVC-associated BSI** | 213 (10.0%) | 203 (8.0%) | 0.017* |
| **CVC- and PVC-associated BSI** | 473 (22.1%) | 472 (18.5%) | 0.002* |
| **NEC** | 136 (6.4%) | 79 (3.1%) | <0.001* |
| **NEC type** |  |  |  |
| No NEC | 2001 (93.6%) | 2467 (96.9%) | <0.001* |
| Surgical NEC | 64 (3.0%) | 44 (1.7%) |  |
| Medical NEC | 37 (1.7%) | 19 (0.7%) |  |
| NEC type unknown | 35 (1.6%) | 16 (0.6%) |  |
| **Time to first NEC [days]** | 21 (11-32) | 16 (10-27) |  |
| **Time from first NEC to end of surveillance [days]** | 33 (5-56) | 47 (19-63) |  |
| **Time to first NEC or discharge** | 52 (38-65) | 50 (37-63) |  |
| **Birth Year** |  |  |  |
| 2004 | 9 (0.4%) | 0 (0.0%) | <0.001* |
| 2005 | 11 (0.5%) | 0 (0.0%) |  |
| 2006 | 120 (5.6%) | 8 (0.3%) |  |
| 2007 | 371 (17.4%) | 11 (0.4%) |  |
| 2008 | 684 (32.0%) | 33 (1.3%) |  |
| 2009 | 633 (29.6%) | 199 (7.8%) |  |
| 2010 | 282 (13.2%) | 513 (20.1%) |  |
| 2011 | 26 (1.2%) | 732 (28.8%) |  |
| 2012 | 1 (0.0%) | 676 (26.6%) |  |
| 2013 | 0 (0.0%) | 355 (13.9%) |  |
| 2014 | 0 (0.0%) | 19 (0.7%) |  |
| **Size of unit [beds]** |  |  |  |
| <20 | 513 (24.0%) | 694 (27.3%) | 0.011* |
| ≥20 | 1624 (76.0%) | 1852 (72.7%) |  |
| **Size of hospital [beds]** |  |  |  |
| < 600 | 821 (38.4%) | 970 (38.1%) | 0.823 |
| ≥ 600 | 1316 (61.6%) | 1576 (61.9%) |  |
| **Neonatal care level** |  |  |  |
| Perinatal center level I | 2123 (99.3%) | 2530 (99.4%) | 0.357 |
| Perinatal center level II | 6 (0.3%) | 11 (0.4%) |  |
| Obstetric clinic | 8 (0.4%) | 5 (0.2%) |  |
| **Type of hospital** |  |  |  |
| University hospital | 824 (38.6%) | 998 (39.2%) | 0.442 |
| Other teaching hospital | 1200 (56.2%) | 1367 (53.7%) |  |
| Other hospital | 113 (5.3%) | 181 (7.1%) |  |
| **Maturity** |  |  |  |
| AGA | 1453 (68.0%) | 1744 (68.5%) | 0.036* |
| SGA | 613 (28.7%) | 716 (28.1%) |  |
| LGA | 60 (2.8%) | 55 (2.2%) |  |
| Missing | 11 (0.5%) | 31 (1.2%) |  |

AGA – Appropriate for gestational age , BSI- blood stream infection, CPAP – Continuous nasal positive airway pressure, CRIB – Clinical risk index for babies, CVC – Central venous catheter, ETT – Endotracheal tube, IQR – interquartile range, LGA – Large for gestational age, Patient days – Total days present on department, PVC – Peripheral venous catheter, Respiratory support includes CPAP and ETT, SGA – Small for gestational age, VC – Venous catheter. Chi-square statistics were performed for categorical variables. * P-values < 0.05 were interpreted as significant.
